# Single‐Cell RNA‐Seq Combined With Bulk RNA‐Seq Revealed the Involvement of Pancreatic Cancer Tissue‐Resident Macrophages in Tumour Progression and the Immunotherapy Response

**DOI:** 10.1111/jcmm.71212

**Published:** 2026-05-28

**Authors:** Bin Wu, Chundong Hu, Fengchun Lu

**Affiliations:** ^1^ The Graduate School of Fujian Medical University Fuzhou China; ^2^ Department of General Surgery Fujian Medical University Union Hospital Fuzhou China; ^3^ Department of Hepatobiliary Surgery The Second Affiliated Hospital of Jiaxing University Jiaxing China

**Keywords:** immunotherapy, pancreatic cancer, single‐cell RNA sequencing, tissue‐resident macrophages, tumour microenvironment

## Abstract

Pancreatic cancer remains a highly lethal malignancy with limited therapeutic efficacy and untapped immunotherapeutic potential, largely constrained by immune cell heterogeneity in the tumour microenvironment. Tumour‐associated macrophages (TAMs), especially tissue‐resident macrophages (TRMs), exert complex regulatory roles in tumour progression. Here, we integrated single‐cell RNA sequencing (scRNA‐seq) and bulk RNA sequencing (bulk‐seq) data from the GEO and TCGA databases to characterize macrophage heterogeneity and its functional impacts in pancreatic cancer. We delineated the tumour microenvironment landscape and identified a specific TRM subpopulation. Cell communication analysis revealed extensive interactions between TRMs and other cell types, including CXCL/MIF and notably upregulated SPP1 signalling in tumour tissues. We further established a TAM scoring system and found that clusters 4, 5, 9 and 10 were significantly associated with patient survival. Among them, TRM cluster 4 showed the highest predictive efficacy for 5‐year and 10‐year mortality, and effectively stratified patients into high‐ and low‐risk groups with distinct differences in survival, immune cell infiltration and immune checkpoint expression. Importantly, TRM_C4 scores exhibited significant changes after immunotherapy, with decreased scores in responders and increased scores in non‐responders. Together, our findings demonstrate the critical involvement of pancreatic cancer tissue‐resident macrophages in tumour progression and immunotherapy response, and suggest that targeting specific macrophage subpopulations may represent a novel strategy to enhance immunotherapy efficacy and improve clinical outcomes for pancreatic cancer patients.

## 1 Introduction

Pancreatic cancer (pancreatic ductal adenocarcinoma, PDAC) is one of the most malignant tumours, with a mortality rate almost equal to its incidence rate [1]. The 5‐year survival rate is only approximately 10%, with some reports indicating that it is less than 5% [1]. Despite continuous breakthroughs and innovations in cancer treatment, satisfactory therapeutic outcomes have not been achieved in pancreatic cancer, unlike in other tumours, and the improvement in prognosis has not been significant [2]. The surgical resection rate of pancreatic cancer is very low, and the efficacy of traditional radiotherapy and chemotherapy is poor. Immunotherapy, which has recently become highly popular, has shown promising results in some tumours, including melanoma, breast cancer, and lung cancer [3]. However, the efficacy of immunotherapy for pancreatic cancer remains uncertain. Recent studies have shown that the tumour microenvironment plays a critical role in tumours, potentially affecting the efficacy of immunotherapy. Researchers believe that the tumour microenvironment can regulate the malignant biological behaviour of tumours; thus, immunotherapy based on the tumour microenvironment is a feasible approach. Therefore, it is necessary to explore the immune microenvironment of pancreatic cancer and the factors that influence it [4].

Recently, the immune microenvironment has been shown to play a crucial role in the progression of pancreatic cancer, with a large extent of macrophage infiltration in tumours being associated with poor clinical outcomes [5]. Tumour‐associated macrophages (TAMs) contribute to tumour progression by promoting angiogenesis, secreting growth factors, and facilitating tumour cell invasion and metastasis [6]. Moreover, TAMs exert immunosuppressive effects, preventing the elimination of tumour cells by natural killer (NK) cells and T lymphocytes [7]. Therefore, targeting the recruitment, survival, and functions of TAMs has become a major therapeutic goal. Although TAMs are considered primarily protumorigenic, several studies have highlighted their protective roles in specific stages of disease and in specific organs [8]. Moreover, different macrophage populations with opposing protumour and antitumour functions may coexist within the same tumour [9]. Consequently, understanding the extent of macrophage heterogeneity is essential for the rational design of macrophage‐targeted therapies.

Macrophage heterogeneity may stem from (1) different states of alternate activation [10], (2) imprinting by tissue or tumour‐derived cues that define macrophage niches [11], (3) different origins of TAMs [12], and (4) systemic modifications induced by tumours in circulating monocytes [13]. In human pancreatic cancer, macrophage infiltration has been assessed via markers such as CD14, CSF1R, and CD68. However, CD14 and CSF1R are also markers for undifferentiated monocytes, and the expression of CD68 in phagocytic cells has not been fully characterized. Other markers, such as CD163, TIE2, MRC1/CD206, and Marco, have been used to evaluate the phenotypic heterogeneity of TAMs. Earlier single‐cell RNA sequencing (scRNA‐seq) studies have challenged the view that alternative activation is the primary cause of TAM heterogeneity. Thus, the phenotypic and functional diversity of TAMs infiltrating human pancreatic tumours remains to be elucidated. Tissue‐resident macrophages (TRMs) are a unique class of immune cells that reside in tissues for extended periods, unlike circulating macrophages [14]. TRMs play important roles in tissue repair, inflammatory responses, and the formation of the tumour microenvironment [15, 16]. The quantity and functional state of TRMs have critical impacts on tumour growth and dissemination [17].

This study aims to leverage scRNA‐seq data to elucidate the heterogeneity and molecular mechanisms of macrophages within the pancreatic cancer microenvironment. An additional aim of this study is the exploration of the relationship between TRMs and tumour progression as well as the immunotherapy response.

## 2 Materials and Methods

### 2.1 Single‐Cell Data Downloading and Processing

The GSE212966 dataset was downloaded and processed with the Seurat package. Cells with fewer than 200 detected genes or a mitochondrial gene content greater than 5% were excluded. After quality control, the Harmony method was used to remove batch effects. Clustering was performed with the parameters npca = 20 and resolution = 0.5 for dimensionality reduction and clustering. To better distinguish the macrophage subpopulations, the parameters npca = 30 and resolution = 0.8 were used for further dimensionality reduction and clustering. The Hallmark gene sets were extracted and downloaded via the msigdbr R package, and their scores were calculated via the GSVA method with the R package GSVA. Data visualization was carried out with the R packages pheatmap and ggplot2. Characteristic genes for each TAM subpopulation were selected with the criterion of logFC > 0.5.

### 2.2 Cell Communication Analysis

Cell communication analysis was conducted using the CellChat package. The CellChatDB.human‐Secreted Signalling database was used for cell interaction analysis, with the gene expression threshold adjusted with the parameter trim = 0.1. Data visualization was performed with functions such as netVisual_circle, netVisual_heatmap, netVisual_aggregate, and netVisual_bubble.

### 2.3 TCGA Data Downloading

The TCGA‐PAAD‐related RNA expression matrix data and clinical data were downloaded with the R package GDCRNATools and standardized. The AnnotationDbi and org.Hs.eg.db databases were used to convert Ensembl IDs to gene symbols in the TCGA dataset, and duplicate gene names were removed.

### 2.4 Calculation of TAM Scores for TCGA Samples

TAM scores for TCGA samples were calculated via the GSVA method with the R package GSVA. The GSVA method measures the expression levels of genes in a gene set relative to the expression levels of all genes via specific statistics such as the Kolmogorov–Smirnov statistic. It ranks the expression values of the genes in each sample and calculates an enrichment score on the basis of the ranking, converting single‐gene expression levels into activity scores for the entire gene set to identify potential differences between sample groups.

### 2.5 Survival Analysis and Construction of the Cox Proportional Hazards Regression Model

Survival analysis and stratification of the TCGA‐PAAD cohort were performed with the survival and survminer packages, with the median TAM score as the cut‐off value (TAM scores greater than the median were considered to indicate high risk, and TAM scores lower than the median were considered to indicate low risk). The Cox proportional hazards regression model was constructed with the coxph function. The univariate Cox model included only a single variable, whereas the multivariate model was adjusted for variables such as sex, age, stage, and race in addition to the TAM score risk. Predicted 5‐ and 10‐year survival rates were analysed and visualized with the pROC package.

### 2.6 Analysis of Immune Cell Infiltration, Immune Function, and Immunotherapy Response

The infiltration of various immune cells was analysed in the TCGA‐PAAD cohort with the R package CIBERSORT, with differences between the high‐ and low‐risk groups analysed by the Wilcoxon test. Microsatellite instability (MSI) scoring was conducted with the BiocOncoTK package. The tumour mutational burden (TMB) and immune phenotype score (IPS) were analysed with the R package TCGAmutations and the IPS_calculation function. Given the lack of publicly available single‐cell RNA‐seq datasets of pancreatic cancer with paired pre‐ and post‐immunotherapy clinical responses, we utilized the GSE123813 dataset (basal cell carcinoma, BCC) as a well‐characterized clinical validation cohort for immunotherapy response analysis. This dataset contains paired single‐cell transcriptomic and T cell receptor sequencing data from patients receiving anti‐PD‐1 therapy with documented clinical response status, enabling reliable evaluation of macrophage subset scores associated with immunotherapy outcomes(Detailed information on the immunotherapy regimen is provided in Table S1). The GSE123813 dataset was downloaded and standardized, and the AddModuleScore function was used to analyse TAM scores on the basis of scRNA‐seq data.

### 2.7 Statistical Analysis

Statistical analyses were performed via R software (version 4.2.1), with *p*‐values less than 0.05 (*p* < 0.05) considered to indicate statistical significance. The following graphing software packages were used in this study: SPSS 22.0, GraphPad Prism 6.02, Adobe Photoshop CS5, and Adobe Illustrator CS6.

### 2.8 Ethics Statement and Informed Consent

This study was conducted in accordance with the Declaration of Helsinki. All data used in this study were obtained from public databases (GEO and TCGA), and no direct human participants were involved. No written consent has been obtained from the patients as there is no patient‐identifiable data included.

## 3 Results

### 3.1 Characterization of the Tumour Microenvironment Landscape and TRM Identification on the Basis of scRNA‐Seq Data

**FIGURE 1:**
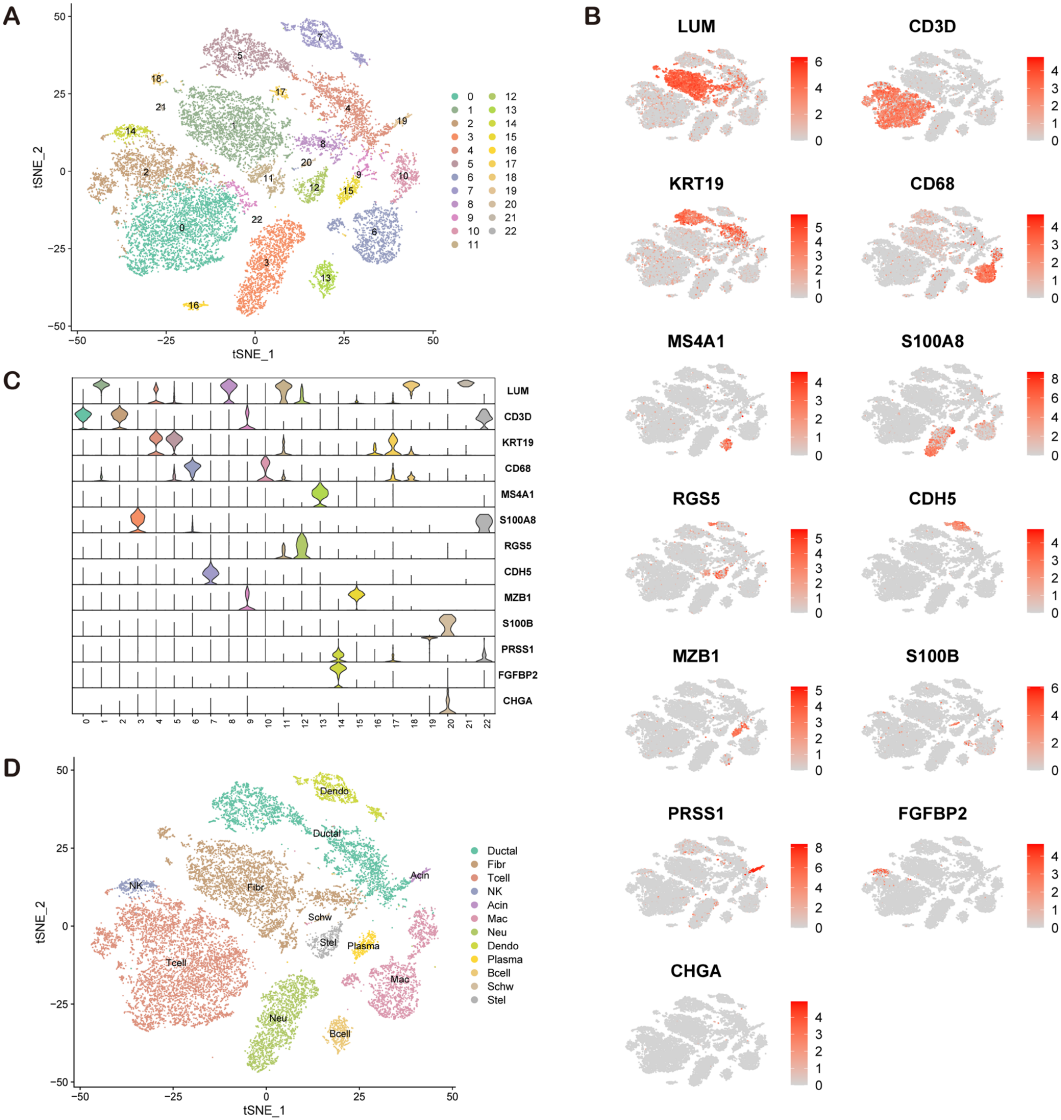
Tumour microenvironment landscape. (A) The 23 clusters identified through unsupervised clustering after removal of batch effects via Harmony. (B, C) Annotation of the cell types, including fibroblasts, T cells, NK cells, B cells, macrophages, and plasma cells, on the basis of common markers. (D) Violin plots showing the distribution and expression levels of signature genes for major cell clusters in the tumour microenvironment.

The GSE212966 dataset was downloaded and thoroughly verified. The integrated data comprised 3 adjacent (ADJ) samples and 6 PDAC samples (another 3 samples had incomplete data and were thus excluded) and included a total of 19,945 cells. After removing batch effects via Harmony, unsupervised clustering was performed, resulting in the identification of 23 clusters (Figure 1A). The cells were annotated on the basis of common markers and identified as fibroblasts, T cells, NK cells, B cells, macrophages, plasma cells, or other types of cells (Figure 1B,C).Figure 1D displays violin plots of signature gene expression levels for each major cell cluster, which supports the reliability of cell clustering and annotation results.

**FIGURE 2:**
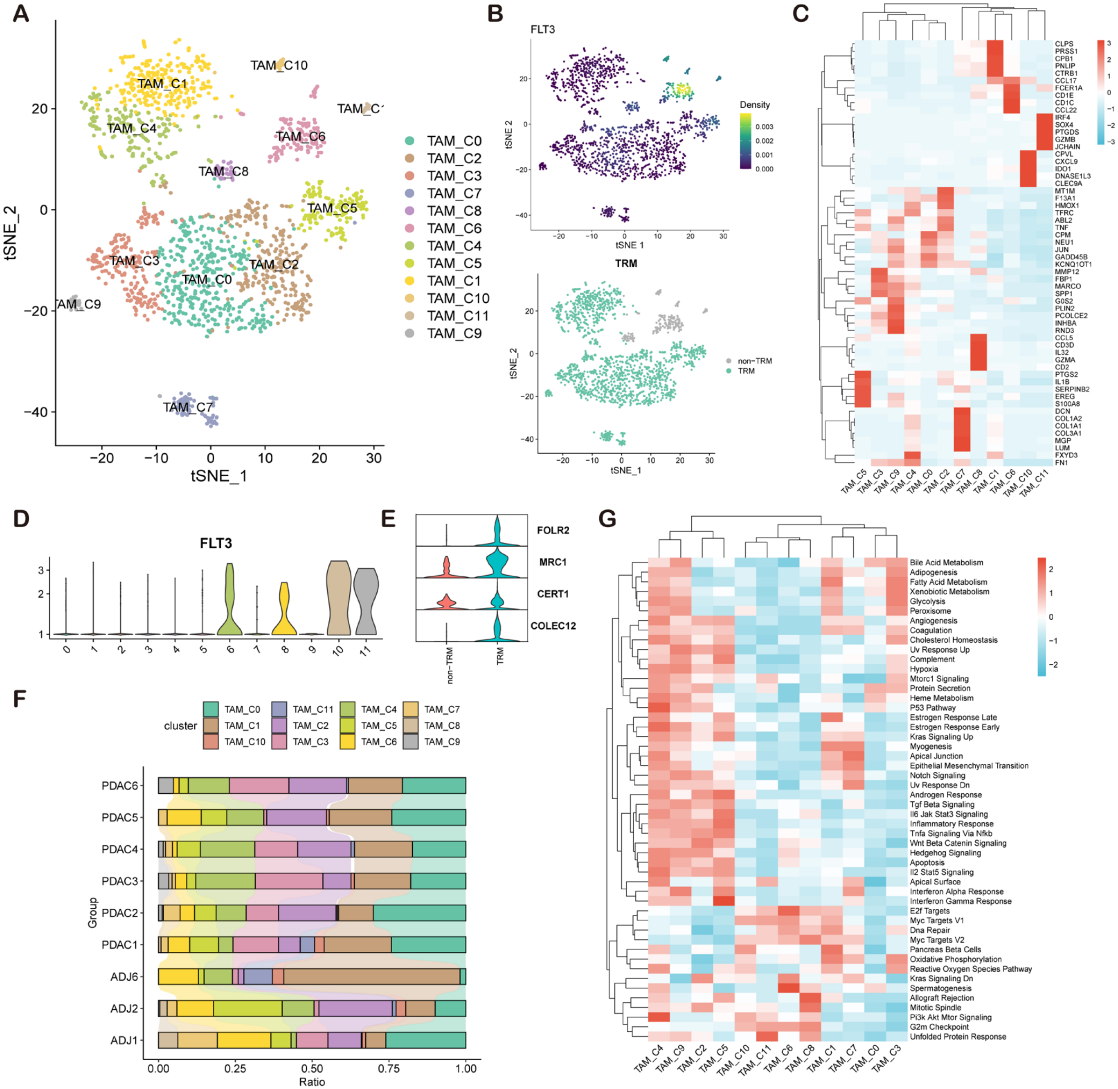
TRM identification and functional analysis of subtypes with the Hallmark gene sets. (A) Clustering analysis of CD68+ macrophages, resulting in the identification of 12 distinct clusters. (B) Clustering on the basis of FLT3 expression to identify subpopulations of macrophages. (C) Heatmap of the top 5 most significantly differentially expressed genes (Top5 genes) in the identified macrophage clusters. (D) Identification of high‐FLT3‐expressing clusters (clusters 6, 8, 10, and 11) as non‐TRM subpopulations. (E) Expression levels of TRM markers in the identified TRM and non‐TRM subpopulations. (F) Frequency analysis showing significant differences in TAM subpopulations between PDAC and ADJ samples. (G) Functional scoring of TAMs via GSVA on the basis of the Hallmark gene sets, highlighting significant functional enhancement in TAM_C4/9/2/5, with activation of pathways such as TGF‐β signalling, IL‐6 JAK‐STAT3 signalling, and inflammation response. Notably, PI3K‐AKT–mTOR activation was particularly prominent in the TAM_C4 cell group.

Macrophages were identified by the expression of CD68. To further distinguish subpopulations of CD68+ macrophages, clustering analysis was performed, resulting in the identification of 12 clusters of macrophages (Figure 2A). A heatmap visualizing the five most significantly differentially expressed genes (Top5 genes) was generated (Figure 2C), and key genes were identified for further subpopulation classification. On the basis of previous literature (PMID: 35690521), 56 genes were used to classify TAMs according to gene sets, namely, IFN‐TAMs, Inflam‐TAMs, LA_TAMs, Angio_TAMs, Reg_TAMs, Prolif_TAMs, and RTM_TAMs, but no significant differences were found [18]. We referenced the study by Baer et al. (Nat Immunol. 2023;24(9):1443–1457. doi: 10.1038/s41590‐023‐01579‐x), in which pancreatic TRMs and macrophages were distinguished in Flt3‐YFP knock‐in mice, with YFP+ macrophages recognized as TRMs. Thus, FLT3 (expressed by bone marrow‐derived monocytes differentiated to macrophages but not by embryonic TRMs) was used to differentiate TRMs from other macrophages [19]. Clustering on the basis of FLT3 expression (Figure 2B) resulted in the identification of clusters 6, 8, 10, and 11 as high‐FLT3‐expressing subpopulations (Figure 2D), which were classified as non‐TRM subpopulations, whereas the remaining FLT3‐ cells were classified as TRM subpopulations. The expression levels of other TRM markers were also significantly increased (Figure 2E). Frequency analysis revealed significant differences in TAM subpopulations between PDAC and ADJ samples (Figure 2F). To further understand the functional differences among TAMs, we used GSVA with the Hallmark gene sets to score the cells (Figure 2G). TAM_C4/9/2/5 were identified as significantly enriched functional cell groups, with pathways such as TGF‐β signalling, IL‐6 JAK‐STAT3 signalling, and inflammation response activated in these groups. Interestingly, PI3K‐AKT–mTOR activation was particularly notable in the TAM_C4 cell group compared with the other cell groups.

### 3.2 Crosstalk Between TRMs and Other Immune Cells

**FIGURE 3:**
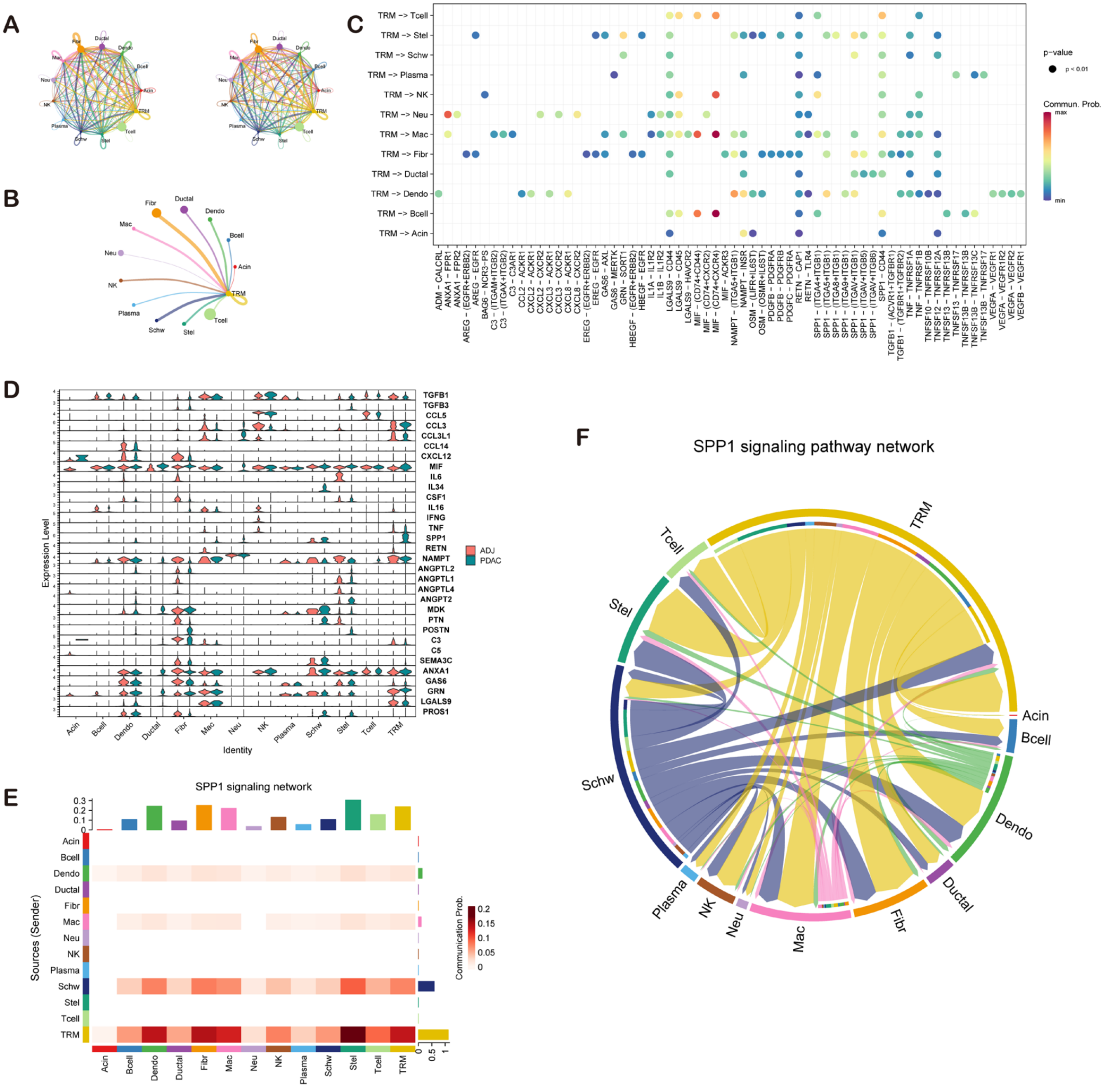
Interaction analysis between TRMs and other cells in the tumour microenvironment. (A–C) Identification of various interaction pathways, including the CXCL/MIF interaction, between TRMs and other cell groups. (D) Differential expression analysis of TRM‐derived signals between PDAC and ADJ samples, highlighting significant differences in TGFB1, CCL3, MIF, SPP1, GRN, and LGALS9 expression, with SPP1 exhibiting the most significant upregulation in PDAC samples. (E) TRMs were identified as the main producers of SPP1 in the tumour microenvironment. (F) Identification of extensive interactions between TRMs and other cells via the SPP1 signalling pathway.

We categorized CD68+ cells as TRMs and non‐TRMs (macrophages, Macs) on the basis of FLT3 expression and mapped all cells according to these classifications for cell communication analysis. We identified multiple forms of interactions, including the CXCL/MIF interaction, between TRMs and various other cell groups (Figure 3A–C). To further explore potential differences, we conducted differential analysis of TRM‐derived signals between PDAC and ADJ samples. Significant differences were found in the expression of TGFB1, CCL3, MIF, SPP1, GRN, and LGALS9, with SPP1 showing the most significant difference and being markedly upregulated in PDAC samples (Figure 3D). Cell communication analysis also revealed extensive interactions between TRMs and other cells via the SPP1 signalling pathway, identifying TRMs as the primary producers of SPP1 (Figure 3E,F).

### 3.3 Association Between TRMs and Clinical Outcomes in Cancer Patients

**FIGURE 4:**
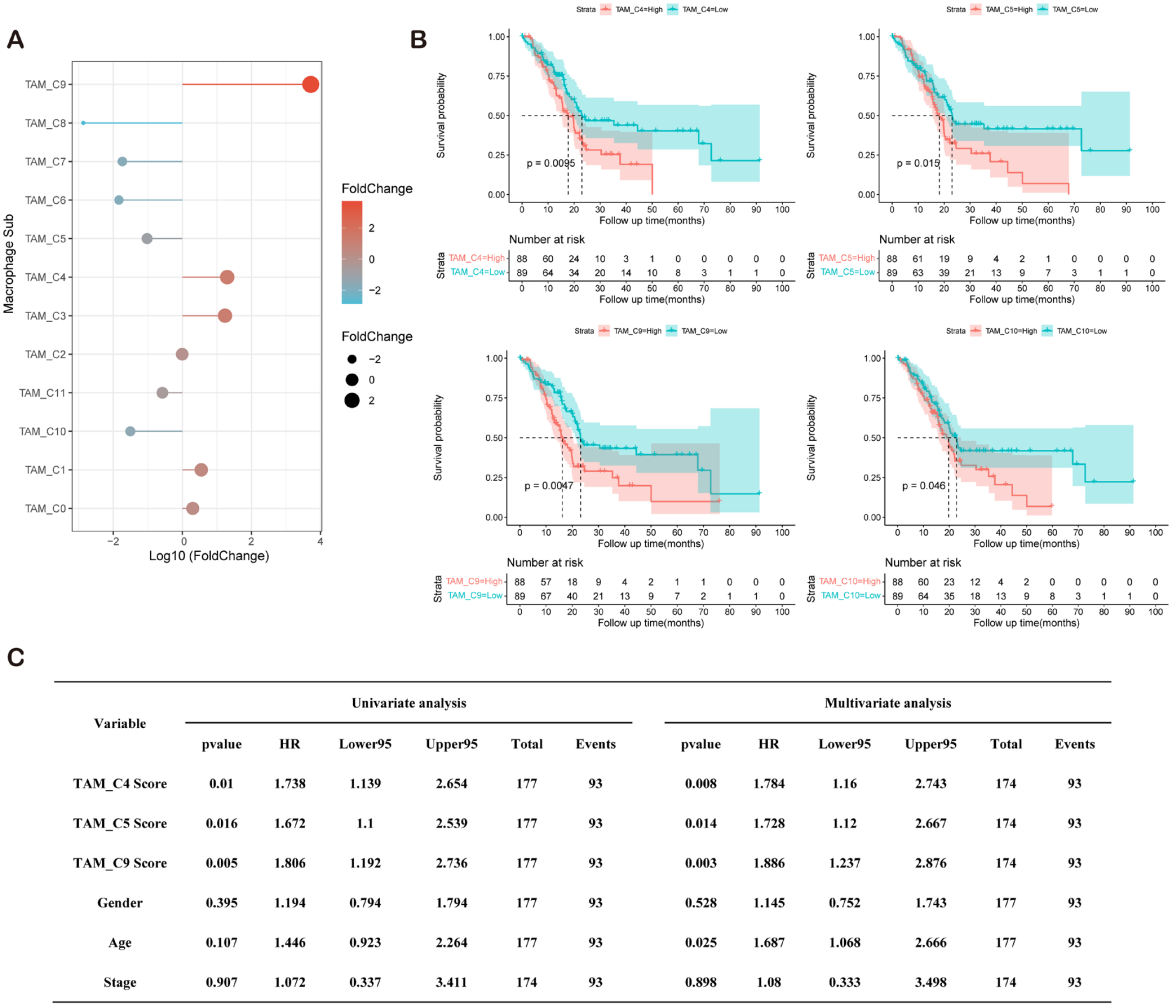
Survival analysis based on the TAM score. (A) Frequency analysis showing significant enrichment of TAM clusters 9, 4, and 3 in PDAC samples. (B) Survival analysis based on the TAM_Score value calculated for each sample using ssgsea, highlighting significant associations between the high‐risk and low‐risk groups stratified on the basis of TAM clusters 4, 5, 9, and 10, most of which were TRM‐related clusters. (C) Univariate and multivariate Cox proportional hazards regression analyses based on TAM clusters 4, 5, and 9 (TRM‐related subpopulations), showing significant associations with disease survival.

We next further explored the relationships between TRM subtypes and disease outcomes. Frequency analysis revealed that TAM clusters 9, 4, and 3 were significantly enriched in PDAC (Figure 4A). Using the FindAllMarkers function (logFC > 0.5; *p* < 0.05), we extracted characteristic genes of all TAMs for subsequent analysis.

We obtained data from 177 pancreatic cancer patients represented in the TCGA database. Using the characteristic genes of the 11 clusters, we calculated a TAM_Score value for each sample with the ssgsea algorithm. The samples were divided into high‐risk and low‐risk groups on the basis of the median TAM score. Survival analysis based on the 11 clusters revealed that TAM clusters 4, 5, 9, and 10—all of which except cluster 10 were TRM clusters—were significantly associated with survival risk (Figure 4B). The mortality risk was positively correlated with the TAM score. Using the Cox proportional hazards model, both univariate and multivariate regression analyses were performed for TAM clusters 4, 5, and 9 (TRM‐related subpopulations), revealing significant associations of these clusters with disease survival (Figure 4C).

**FIGURE 5:**
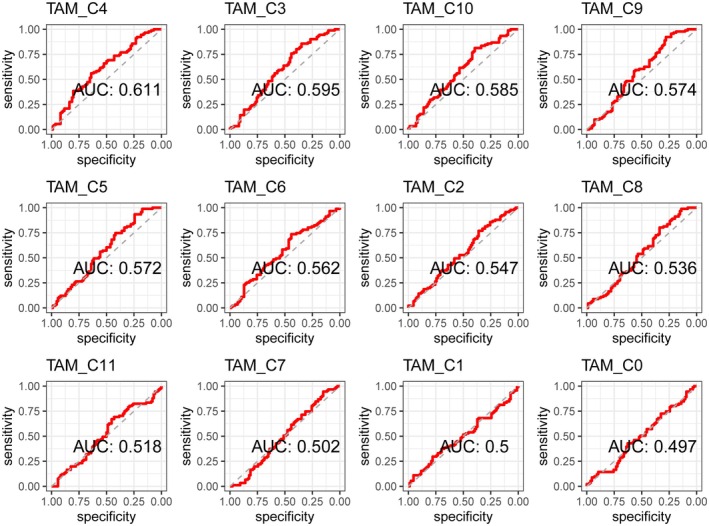
Receiver operating characteristic (ROC) curves for the prediction of 5‐year mortality based on the TAM subtype score. This figure shows the ROC curves for the prediction of 5‐year mortality rates based on the scores of various TAM clusters. Compared with the other clusters, TRM cluster 4 demonstrated the highest predictive efficacy, with an AUC of 61.1%.

**FIGURE 6:**
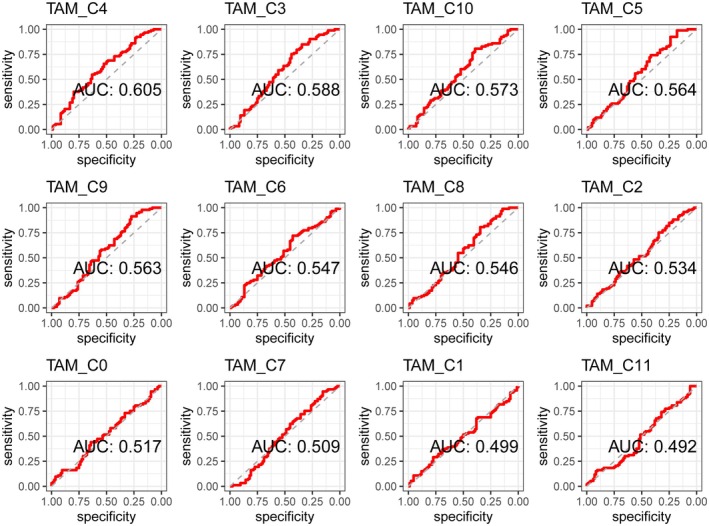
ROC curves for the prediction of 10‐year mortality based on the TAM subtype score. This figure shows the ROC curves for the prediction of 10‐year mortality rates based on the scores of various TAM clusters. Compared with the other clusters, TRM cluster 4 had the highest predictive efficacy, with an AUC of 60.5%.

Then, using these scores, we conducted a predictive analysis of 5‐year and 10‐year mortality in patients. Among the clusters, TRM cluster 4 demonstrated the highest predictive efficacy. The area under the curve (AUC) for 5‐year mortality prediction was 61.1% (Figure 5). The AUC for 10‐year mortality prediction was 60.5% (Figure 6).

### 3.4 Correlations of the TRM Score With Immune Cells, Immune Function, and the Immunotherapy Response

**FIGURE 7:**
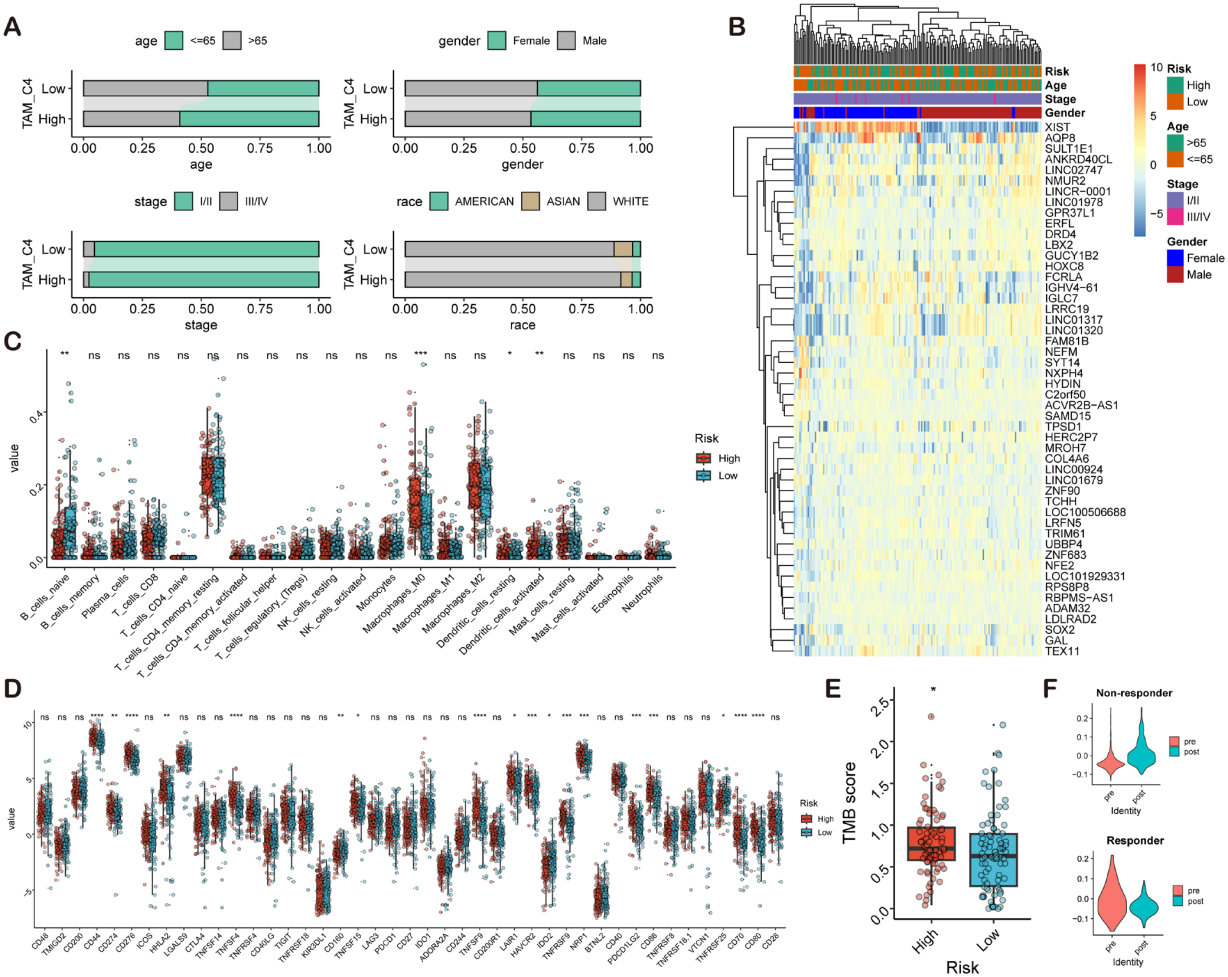
Clinical and pathological characteristics and prospective treatment outcomes of patients in the high‐risk and low‐risk groups based on TAM_C4 as a TRM subpopulation. (A,B) Differential gene expression analysis between high‐risk and low‐risk PAAD patients, showing significant differences in gene expression profiles. (C) Immune cell analysis highlighting the lower abundances of naive B cells and both resting and activated dendritic cells but greater abundance of M0 macrophages in high‐risk patients. (D) Abnormally high expression of various immune checkpoints, including PD‐1 (CD274), in high‐risk patients. (E) Higher TMB in high‐risk patients, with no correlations found with other scores. (F) Differences in the TAM_C4 score between treatment responders (*n* = 9) and nonresponders (*n* = 2), with nonresponders showing increased posttreatment scores and responders showing decreased posttreatment scores.

We analysed the clinical characteristics associated with high and low TRM_C4 risk scores, revealing distinct clinical distribution patterns. High‐risk scores were more prevalent among patients aged 65 years or younger, female patients, individuals with type I/II classification, and white patients. Differential gene expression analysis between high‐risk and low‐risk PAAD patients also revealed significant differences between these patient groups (Figure 7A,B). Immune cell infiltration analysis, the TMB/MSI/IPS scores, and immune checkpoint analysis indicated that high‐risk patients had lower abundances of naive B cells and both resting and activated dendritic cells but a greater abundance of M0 macrophages (Figure 7C). High‐risk patients had higher TMBs, while no correlations were found with other scores (Figure 7E). Additionally, high‐risk patients had abnormally high expression of various immune checkpoints, including PD‐1 (CD274) (Figure 7D). These results suggest that Cluster 4‐based scoring can be used to classify patients effectively into high‐risk and low‐risk groups, with significant differences in survival rates, immune cell infiltration, and immune checkpoint expression between the groups. Furthermore, we found that the TAM_C4 score differed significantly between treatment responders and nonresponders; nonresponders had increased posttreatment scores, whereas responders had decreased posttreatment scores (Figure 7F). Using a validated anti‐PD‐1 clinical cohort (GSE123813), these findings indicate that TRMs, particularly those in the cluster 4 subpopulation, play crucial roles in the pathogenesis of cancer and may have significant implications for patient outcomes and the response to immunotherapy.

## 4 Discussion

Macrophages were initially thought to be sensors of pathogen infections and tissue damage, and recent studies have expanded our understanding by identifying a type of macrophage originating from the embryo and conserved in vertebrates [20, 21]. These macrophages, known as resident macrophages, persist in the human body and maintain stable, close contacts with specific tissue cells. Resident macrophages exist in nearly all tissues and are endowed with some tissue‐specific physiological functions. Resident macrophages sense changes in environmental signals [22] and mediate the growth, remodelling, and balance of tissue cells. In both mice and humans, genetic abnormalities that impair macrophage function lead to severe developmental disorders, including delayed neurodevelopment; skeletal deformities; impaired tissue repair and remodelling; and dysfunction of the liver, spleen, reproductive system, lungs, and cardiovascular system [23]. Recently, substantial advancements in fate‐mapping models, transcriptomics, and epigenetics have allowed researchers to more easily describe the precise localization and developmental origins of macrophages. These tools have also facilitated our understanding of the regulatory roles of macrophages in development, degenerative diseases, tumours, and inflammatory diseases. Although TAMs play complex roles in tumour tissues, different subgroups of cells exposed to different tissue environments have different functions, and scRNA‐seq data have made it possible to comprehensively map and study the various cells in tumours and their phenotypic functions.

In the present study, we characterized the landscape of the tumour microenvironment of pancreatic cancer via analysis of scRNA‐seq data. Macrophages were further subdivided into 12 clusters based on CD68 expression. We used FLT3‐CD68+ cells to distinguish TRM cells. We identified the TAM_C4/9/2/5 clusters as significantly functionally enhanced cell groups, with activity of the TGF‐β signalling pathway, IL‐6 JAK‐STAT3 signalling pathway, and inflammation response pathway in these clusters. Further analysis revealed extensive crosstalk between TRMs and various cell types. Notably, among the genes involved in TRM crosstalk, SPP1 presented the most significant differential expression between PDAC and ADJ tissues. This finding is consistent with previous research indicating that mTORC2‐deficient macrophages undergo proinflammatory reprogramming and promote inflammation and tumour growth via the cytokine SPP1/osteopontin [24]. Consistent with our observation of elevated SPP1 expression in PDAC(Figure 3D), accumulating evidence has established SPP1, also known as osteopontin, as a multifunctional regulator of pancreatic cancer progression that shapes a protumorigenic and immunosuppressive microenvironment [25]. SPP1 is highly expressed in tumour cells, cancer‐associated fibroblasts, and tumour‐associated macrophages, and its upregulation correlates with advanced tumour stage, distant metastasis, and poor overall survival in PDAC patients [26]. Notably, SPP1 is increasingly recognized as a hallmark signature of a functionally distinct subset of macrophages in pancreatic cancer. Within the PDAC microenvironment, macrophages acquire an SPP1^+^ phenotype under the influence of hypoxia and tumour‐derived cytokines, thereby shifting toward an immunosuppressive and profibrotic state [27, 28]. Our identification of SPP1 upregulation in PDAC therefore carries important biological implications beyond descriptive profiling: it highlights SPP1 as a key molecular node connecting TRM dysfunction, immune suppression, and adverse clinical outcomes. Taken together with previous literature, our results support that SPP1 is not only a prognostic indicator but also a potential therapeutic target to reverse immunosuppression and impede PDAC progression.

In the TAM score‐based survival analysis, the TAM scores of clusters 4, 5, 9, and 10—all of which except cluster 10 were TRM clusters—were significantly associated with survival risk. Predictive analysis of mortality based on the TAM score revealed an AUC of 61.1% for 5‐year mortality and 60.5% for 10‐year mortality. High risk scores for the key cell subpopulation TRM_C4 were more prevalent among patients aged 65 years or younger, female patients, individuals with type I/II classification, and white patients. High‐risk patients had lower abundances of naive B cells and both resting and activated dendritic cells but a greater abundance of M0 macrophages. High‐risk patients also exhibited a higher TMB and abnormally high expression of multiple immune checkpoints, including PD‐1 (CD274). There were significant differences in TAM_C4 scores between responders and nonresponders before and after treatment. Nonresponders had increased posttreatment scores, whereas responders had decreased posttreatment scores. These findings suggest that TRMs play a crucial role in the pathogenesis of pancreatic cancer, with the cluster 4 subpopulation potentially playing an important role.

Recent studies have provided insights into the potential role of the PI3K‐AKT–mTOR pathway in regulating tissue‐resident macrophage (TRM) function. For instance, alveolar macrophages, a type of TRM, exhibit increased glucose responsiveness outside the lung environment, leading to upregulation of glycolysis and activation of the PI3K‐Akt, mTOR, and C/EBPb pathways [29]. Additionally, research on peritoneal TRMs has shown that Gata6 deficiency leads to enhanced oxidative phosphorylation and alternative activation, which may be linked to metabolic reprogramming involving these pathways [30]. Furthermore, FOLR2+ TRMs in breast cancer, which are associated with CD8+ T cell infiltration and potentially anti‐tumour activity, might also utilize this pathway for their functional regulation, although direct evidence is still lacking [31]. These findings collectively suggest that the PI3K‐AKT–mTOR pathway is a critical regulator of TRM phenotype and function across different tissues. Elaborating on how this pathway specifically governs the unique properties of TRM_C4 would significantly enhance the mechanistic clarity of its pronounced clinical associations.

There is increasing recognition of the importance of TAMs in tumour therapy. Studies have shown that CSF‐1 receptor inhibitors can effectively inhibit the proliferation of endometrial cancer (EC) cells and macrophage infiltration. CSF‐1 and its receptor are highly expressed in EC tissues and the ECC‐1 and HEC‐1A cell lines. Wound healing and chemotactic migration assays demonstrated that EC cell supernatant promoted the migration of the U937 macrophage line, but this migration ability was reduced after CSF‐1R blockade. Further studies indicated that inhibiting CSF‐1 expression in EC cells also limited U937 cell migration. Additionally, treating U937 cells with PLX3397 inhibited the proliferation of EC cells by reducing the expression of various proliferation‐related proteins, such as Janus kinase‐1, phosphoinositide 3‐kinase, AKT, cyclin‐dependent kinases 2 and 4, and retinoblastoma‐related protein. These results indicate that CSF‐1 not only promotes macrophage migration but also increases EC cell proliferation through CSF‐1‐stimulated macrophages, highlighting the critical role of CSF‐1 and its receptor in driving macrophage infiltration and EC progression [32]. Another study explored how interactions between TAMs and cancer cells affect epithelial–mesenchymal transition (EMT) and circulating tumour cells (CTCs) in colorectal cancer (CRC) and examined the role of this process in CRC metastasis. The results revealed that CD163+ TAMs at the invasive front are closely associated with EMT, mesenchymal CTC proportions, and poor outcomes in CRC patients. In vitro experiments demonstrated that TAMs secrete IL‐6, activating the JAK2/STAT3 signalling pathway and inducing EMT in CRC cells, thereby increasing their migration and invasion capabilities. Furthermore, IL‐6 signalling indirectly increased the expression of the transcription factor FoxQ1 by inhibiting miR‐506‐3p, promoting CCL2 production and increasing macrophage recruitment. These findings reveal the existence of a positive feedback loop between TAMs and CRC cells, where TAMs promote CRC metastasis by regulating EMT. These findings provide new insights into the role of TAMs in the CRC microenvironment and offer a theoretical basis for developing therapies targeting CRC metastasis [33].

Liu X and his team employed scRNA‐seq and bulk sequencing (bulk‐seq) techniques to perform an in‐depth analysis of CRC patients, focusing on intercellular communication, immune infiltration, and macrophage subpopulation characteristics. They identified seven distinct macrophage subpopulations and revealed that IDO1 macrophages exhibit infiltration patterns that varied with CRC characteristics. By analysing 12 key marker genes of IDO1 macrophages, patients were classified into the high‐IDO1 macrophage (H‐IDO1M) and low‐IDO1 macrophage (L‐IDO1M) groups. The H‐IDO1M group displayed greater immune cell infiltration, greater immune checkpoint expression, and lower pathological stage. These findings suggest that the IDO1 macrophage subtype can predict the patient response to immunotherapy and has important clinical implications for the development of effective immunotherapy strategies. That study highlights the potential of using IDO1 macrophages as biomarkers to predict the immunotherapy response and underscores the importance of macrophage classification in cancer therapy design [34]. However, other studies suggest that dense macrophage infiltration at the tumour front has a positive effect on CRC prognosis and that the extent of cell–cell contact may influence the balance between the protumour and antitumour properties of macrophages. In vitro experiments revealed that when the ratio of macrophages to CRC cells is high, macrophages can inhibit cancer cell growth, a process that is partially dependent on direct cell‐to‐cell contact. In the absence of such contact, macrophages promote cancer cell proliferation, although this study did not distinguish between macrophage subpopulations [35]. In breast cancer, immune cells, particularly macrophages, are most densely infiltrated at the invasive front of the tumour, with the greatest TGF‐β signalling activity in these cells. These findings indicate that the progression and aggressiveness of human breast cancer are closely related to collagen linearization and stromal stiffening, which involve tissue inflammation and the TGF‐β signalling pathway [36].

Research on TRMs in tumours is still limited. In one breast cancer study, TRM clustering was utilized to generate a 25‐gene expression signature (RTM.Sig) for predicting the immunotherapy response. Validation with bulk‐seq datasets from breast cancer samples revealed that RTM.Sig accurately predicted immunotherapy outcomes, with greater predictive accuracy than previously reported ICT response signatures. These findings not only enhance our understanding of the role of TRMs in breast cancer but also potentially improve clinical diagnostic and treatment strategies, providing more effective tools for immunotherapy decision making for breast cancer patients [37]. Regarding the spatial localization of macrophages, extensive immunolabelling of human tissues and online analysis of cancer transcriptomic datasets revealed high expression of TIM4 in specific macrophage populations located in T‐cell regions of tumour‐associated tertiary lymphoid structures (TLSs). TIM4 expression was positively correlated with B‐cell marker expression, effector CD8+ T cells, and the expression of 12 chemokine markers for TLSs. TLS macrophages expressing TIM4 and FOLR2 (TLSTIM4 + Mφ) were enriched in cancers with MSI and abundant CD8+ T‐cell infiltration, indicating their association with immune‐active tumours. These macrophages differed in their expression of immune inhibitory molecules; CATIM4 + Mφ expressed higher levels of TREM2, IL10, and TGFβ than TLSTIM4 + Mφ did. scRNA‐seq analysis of tumour‐associated myeloid cells revealed two TIM4 + FOLR2+ clusters consistent with CATIM4 + Mφ and TLSTIM4 + Mφ, each defined by specific gene signatures. The signature of CATIM4 + Mφ was correlated with poorer patient survival, whereas the gene signature of TLSTIM4 + Mφ was associated with better prognosis. These data suggest that TIM4 is a marker for two distinct macrophage populations with different phenotypes and tissue localization characteristics that potentially play opposite roles in tumour immunity [38]. Another study examined the antitumour role of macrophages in tumour tissues. This study investigated how influenza virus (IAV) infection results in the training of alveolar macrophages (AMs) residing in the respiratory mucosa, endowing them with long‐term antitumour immune functions. A study in a mouse model revealed that IAV‐trained AMs could provide long‐term, tumour‐specific immune protection. These trained AMs infiltrated lung tumour lesions, exhibited augmented phagocytic and tumoricidal functions, and were resistant to tumour‐induced immunosuppression. This trained antitumour immunity depended on interferon‐gamma (IFNγ) and NK cells. Notably, a favourable immune microenvironment, associated with human AMs possessing antitumour immune characteristics, was observed in non‐small cell lung cancer (NSCLC) tissue. These findings reveal the role of trained resident macrophages in pulmonary mucosal antitumour immune surveillance and suggest that inducing trained immunity in resident macrophages could be a durable antitumour strategy.

Another study revealed that TRMs with high CD163 expression (CD163^hi macrophages) play a crucial role in resistance to T‐cell‐based immunotherapy, showing resistance to both primary and secondary treatments. These CD163^hi M2 macrophages, located at the invasive margin of the tumour, exhibited strong resistance to Csf1r‐targeted therapy and affected T‐cell infiltration in the tumour. This study proposed new therapeutic strategies targeting these macrophage subpopulations to overcome immunotherapy resistance [39]. Additionally, a study published in July 2020 explored the role of M1^hot TAMs in human lung cancer. These cells promoted the infiltration and survival of tissue‐resident memory T (T_RM) cells. Detailed transcriptomic analysis of early‐stage lung cancer patients revealed that M1^hot TAMs augment T‐cell responses and improve patient survival outcomes by expressing specific chemokines such as CXCL9. This research underscores the therapeutic potential of modulating TAM phenotypes to increase immune responses [40].

Among previous studies on TAMs in pancreatic cancer, one study examined the mechanisms of resistance to chemotherapy in proliferating TRMs (proliferating rM4s) in PDAC. In that study, scRNA‐seq, multicolour immunohistochemistry, flow cytometry, and metabolomic analyses revealed that these macrophages reduce gemcitabine uptake by increasing deoxycytidine (dC) production and decreasing dC kinase (dCK) expression, thereby promoting chemoresistance, fibrosis, and immunosuppression. Experiments showed that the elimination of these macrophages alleviated fibrosis and immunosuppression and restored chemosensitivity, providing new therapeutic strategies for PDAC [11]. Another review discussed the role of macrophages in the tumour microenvironment, particularly the different functions of tissue‐resident and recruited macrophages at primary and metastatic sites. This review highlighted macrophage heterogeneity and its crucial roles in regulating tumour progression, immune responses, and therapy responses, providing an overview of therapeutic strategies targeting macrophages, including methods for depletion, recruitment inhibition, and reprogramming (11). The role of CXCR4+ macrophages in PDAC progression has been extensively studied. Abundant infiltration of these macrophages is significantly associated with poor prognosis in PDAC and involves extracellular matrix remodelling and cell proliferation through the CXCR4/PI3K/Akt pathway. CXCR4+ macrophage infiltration is an indicator of poor prognosis in PDAC, suggesting that targeting CXCR4+ macrophages could be crucial for a favourable response to PDAC immunotherapy [41].

## 5 Conclusions

In this study, scRNA‐seq analysis was utilized to explore the complex landscape of the pancreatic cancer tumour microenvironment, revealing significant heterogeneity among macrophage populations. Our findings emphasize the critical roles of TRMs and TAMs in regulating tumour progression and the response to immunotherapy. Specifically, we identified particular macrophage subpopulations associated with either protumour or protective functions, highlighting the dual roles that these cells can play within the same tumour microenvironment. Notably, proliferating rM4s demonstrated a mechanism of chemoresistance in PDAC through the modulation of the tumour metabolic environment, a mechanism that can be targeted to increase the efficacy of chemotherapy.

Extensive intercellular communication analysis revealed complex interactions between TRMs and other cellular components within the tumour microenvironment, providing potential therapeutic targets. Our data suggest that modulating the signalling pathways and interaction networks of TRMs and TAMs could shift the tumour microenvironment toward a more immunostimulatory state, potentially improving patient outcomes. Furthermore, the correlation between TAM heterogeneity and patient survival, as well as the unique characteristics of TAM subtypes, offers deeper insights into the clinical significance of TAMs. By integrating single‐cell lineage data with bulk‐seq data, we provided a comprehensive perspective on immune dynamics in pancreatic cancer, supporting the development of more precise and effective macrophage‐targeted therapeutic strategies.

Overall, this study not only advances our understanding of the immunological foundations of pancreatic cancer but also paves the way for improving immunotherapy approaches by targeting specific macrophage populations within the tumour microenvironment. Further research and clinical trials will be crucial to validate these findings and translate them into effective treatment modalities for pancreatic cancer patients.

## Author Contributions

**Bin Wu:** conceptualization, funding acquisition, methodology, data curation, formal analysis, writing – original draft, writing – review and editing. **Chundong Hu:** data curation, validation, formal analysis, writing – original draft, visualization. **Fengchun Lu:** conceptualization, funding acquisition, writing – review and editing, project administration.

## Funding

This work was supported by Project of Starlit South Lake Leading Elite Program, 2023AD400006. National Natural Science Foundation of China, 82073139. Fujian Provincial Natural Science Foundation of China, 2020J02054. Joint Funds for the Innovation of Science and Technology, Fujian Province, 2021Y9058.the Bureau of Science and Technology of Jiaxing City, 2024AD30085.

## Ethics Statement

This study was approved by the ethics committee of the Second Affiliated Hospital of Jiaxing University, Jiaxing, China.

## Consent

All authors gave their consent for publication.

## Conflicts of Interest

The authors declare no conflicts of interest.

## Supporting information


**Table S1:** Clarification of Immunotherapy Details.

## Data Availability

The data that support the findings of this study are available on request from the corresponding author. The data are not publicly available due to privacy or ethical restrictions.
