# Supplementary material for: Single‐Cell RNA‐Seq Combined With Bulk RNA‐Seq Revealed the Involvement of Pancreatic Cancer Tissue‐Resident Macrophages in Tumour Progression and the Immunotherapy Response
Source: J Cell Mol Med. 2026 May 28;30(11):e71212. doi: 10.1111/jcmm.71212 (PMC13240574; doi:10.1111/jcmm.71212)
Supplement: Supplementary file 1 — Table S1: Clarification of Immunotherapy Details. [file JCMM-30-e71212-s001.docx]

Table S1 **Clarification of Immunotherapy Details**

| **Item** | **Details** |
| --- | --- |
| Immunotherapy Type | PD‑1 checkpoint blockade   1. Pembrolizumab (anti‑PD‑1) 2. Cemiplimab (anti‑PD‑1) 3. Vismodegib (Hedgehog pathway inhibitor, not immunotherapy) for a subset of patients |
| Treatment Regimen | 1. Pembrolizumab:200 mg intravenously every 3 weeks 2. Cemiplimab:350 mg intravenously every 2 weeks 3. Vismodegib:150 mg orally once daily (for some patients) |
| Patient Inclusion Criteria | 1. Histologically confirmed advanced basal cell carcinoma (BCC) or squamous cell carcinoma (SCC) 2. Not suitable for surgical resection. 3. No prior immune checkpoint inhibitor therapy. 4. No systemic immunosuppressants within 4 weeks before the first biopsy.   5. No radiotherapy or other anticancer therapy within 4 weeks before the first biopsy |
| Response Evaluation Criteria | Tumor response was assessed using **RECIST version 1.1** |
